# Supplementary material for: Generation of Functional Immortalized Human Corneal Stromal Stem Cells
Source: Int J Mol Sci. 2022 Nov 2;23(21):13399. doi: 10.3390/ijms232113399 (PMC9657819; doi:10.3390/ijms232113399)
Supplement: Supplementary file 1 [file ijms-23-13399-s001.zip › ijms-1900916-supplementary.pdf]

Table S1: List of primer pairs used for RT-PCR.

Gene ID (name)	Forward 5'-3'	Reverse 5'-3'
**Adipogenic markers**		
FABP4	ATGGGATGGAAAATCAACCA	GTGGAAGTGACGCCTTTCAT
PLIN	AAACAGCATCAGCGTTCCCCA	AGTGTTGGCAGCAAATTCCG
**Osteogenic markers**		
ALP	TACAAGGTGGTGGGCGGTGAACGA	TGGCGCAGGGGCACAGCAGAC
SPARC	GGCATCAAGCAGAAGGAT	GCACCGTTAATGTATTCACT
**Chondrogenic markers**		
ACAN	AGCCTGCGCTCCAATGACT	TAATGGAACACGATGCCTTTCA
COMP	AGCAGATGGAGCAAACGTATTG	ACAGCCTTGAGTTGGATGCC
**Keratocyte-lineage markers**		
LUM	CCTGGTTGAGCTGGATCTGT	TGGTTTCTGAGATGCGATTG
KERA	ATCTGCAGCACCTTCACCTT	CATTGGAATTGGTGGTTTGA
PTGDS	CGGGGTCCCTCGGCTCCTAC	CTGGGGGTCTGGGTTCGGCT
PAX6	TGGGCAGGTATTACGAGACTG	ACTCCCGCTTATACTGGGCTA
**TSG-6 transcript**		
TNFAIP6	AAGCACGGTCTGGCAAATACAAGC	ATCCATCCAGCAGCACAGACATGA
**Housekeeping genes**		
18S	CCCTGTAATTGGAATGAGTCCAC	GCTGGAATTACCGCGGCT
GAPDH	ACAACTTTGGTATCGTGGAAGG	GCCATCACGCCACAGTTTC
